# Single Nucleotide Polymorphism (SNP) markers associated with high folate content in wild potato species

**DOI:** 10.1371/journal.pone.0193415

**Published:** 2018-02-23

**Authors:** Sapinder Bali, Bruce R. Robinson, Vidyasagar Sathuvalli, John Bamberg, Aymeric Goyer

**Affiliations:** 1 Hermiston Agricultural Research and Extension Center, Oregon State University, Hermiston, OR, United States of America; 2 Department of Crop and Soil Science, Oregon State University, Corvallis, OR, United States of America; 3 USDA/Agricultural Research Service, US Potato Genebank, Sturgeon Bay, WI, United States of America; 4 Department of Botany and Plant Pathology, Oregon State University, Corvallis, OR, United States of America; USDA/ARS, UNITED STATES

## Abstract

Micronutrient deficiency, also known as the hidden hunger, affects over two billion people worldwide. Potato is the third most consumed food crops in the world, and is therefore a fundamental element of food security for millions of people. Increasing the amount of micronutrients in food crop could help alleviate worldwide micronutrient malnutrition. In the present study, we report on the identification of single nucleotide polymorphism (SNP) markers associated with folate, an essential micronutrient in the human diet. A high folate diploid clone Fol 1.6 from the wild potato relative *Solanum boliviense* (PI 597736) was crossed with a low/medium folate diploid *S*. *tuberosum* clone USW4self#3. The resulting F_1_ progeny was intermated to generate an F_2_ population, and tubers from 94 F_2_ individuals were harvested for folate analysis and SNP genotyping using a SolCap 12K Potato SNP array. Folate content in the progeny ranged from 304 to 2,952 ng g^-1^ dry weight. 6,759 high quality SNPs containing 4,174 (62%) polymorphic and 2,585 (38%) monomorphic SNPs were used to investigate marker-trait association. Association analysis was performed using two different approaches: survey SNP-trait association (SSTA) and SNP-trait association (STA). A total of 497 significant SNPs were identified, 489 by SSTA analysis and 43 by STA analysis. Markers identified by SSTA were located on all twelve chromosomes while those identified by STA were confined to chromosomes 2, 4, and 6. Eighteen of the significant SNPs were located within or in close proximity to folate metabolism-related genes. Forty two SNPs were identical between SSTA and STA analyses. These SNPs have potential to be used in marker-assisted selection for breeding high folate potato varieties.

## Introduction

Micronutrient malnutrition is a global health concern that affects as many as two billion people [1, 2]. Health problems associated with malnutrition are responsible for over a million deaths per year [3]. Folate (a.k.a. vitamin B_9_) is an essential micronutrient in the human diet. Folate plays an important role in overall cellular and organismal health. In the absence of sufficient folate intake, cellular processes such as nucleic acid biosynthesis, the metabolism and catabolism of amino acids, and the methylation cycle cannot take place efficiently [4]. Folate deficiencies have been linked to many serious health concerns such as congenital birth defects, anemia, increased risk of stroke, certain types of cardiovascular diseases and cancers [5–8]. Neural tube defects (NTDs) such as spina bifida and anencephaly are some of the most common congenital birth defects, with an estimated 250,000 cases of NTDs worldwide [9]. It is estimated that up to 70% of NTDs can be prevented with proper folate intake or folate supplementation [6]. Low folate levels have also been linked to impaired cognitive performance and depression [10–12].

The cultivated potato (*Solanum tuberosum* L.) is one of the most consumed food crops worldwide, with a total world production of over 382 million tons in 2014, following maize, rice, and wheat (FAOSTAT data). Potatoes are cultivated in over one hundred countries, from sea level up to 4,700 meters above sea level. It is estimated that over one billion people worldwide consume potatoes regularly [13] which makes potatoes a fundamental element of food security for millions of people.

Biofortification of potatoes for increased folate content could be an efficient strategy to reduce folate deficiencies around the world. Potato has a tremendous genetic diversity with over 4,000 varieties and ~100 wild related species [14–17]. This biodiversity represents a tremendous resource to search for traits of interest such as high folate content. Systematic screening of potato germplasm for tuber folate content has shown a 10-fold range of folate concentrations with some accessions of wild species containing up to 4-fold the folate content of modern potatoes [18, 19].

Selecting for high folate content in potato tubers is a tedious process because potatoes must go through a full growing season to obtain tubers for testing, and it takes approximately three days to determine folate content for every eighteen to twenty samples, making large-scale selection for high folate trait practically impossible. Screening efficiency would be greatly improved by the development and use of molecular markers associated with high folate content. Tools such as SNP genotyping, whole genome sequencing, quantitative trait loci (QTL) analysis, and marker-trait associations are useful for the observation and mapping of genetic differences between breeding clones and the progeny of crosses, as well as the contributions of these differences to the coding regions of genes for specific traits [15, 20–22].

Molecular markers based genetic linkage maps for diploid and tetraploid potatoes have been constructed previously [23–25]. Association mapping, commonly known as linkage disequilibrium (LD)-based mapping is becoming extremely popular for the identification of QTL for investigating marker-trait associations in plants. This strategy serves as an effective alternative to the linkage-based traditional approaches and offers an advantage to breeders, making the requirement of inbred crosses unnecessary [26]. Association mapping has been reported for various phenotypic/morphological traits in potatoes [27–30]. These tools can make introgression of beneficial traits into potatoes more efficient and breeders can improve breeding efficiency by selecting individuals based on a comprehensive set of genetic data associated with desired traits (e.g., disease resistance, drought tolerance, skin/flesh color, and nutritional content). Theoretically, marker assisted selection (MAS) in potatoes can substantially reduce the amount of time it takes for breeders to develop superior breeding clones for commercial use, licensing and release.

The primary purpose of this study was to identify molecular markers associated with high folate content in potato tubers. We used association mapping to find marker trait associations and identify SNPs associated with high folate content in potato for their potential use in marker assisted breeding.

## Materials and methods

### Plant material

High folate values were previously reported in bulked tubers from four seedlings of the *Solanum boliviense* PI 597736 accession [18]. Fine screening showed that this accession segregated for folate. A clone referred to as Fol 1.6 that had high tuber folate content was crossed with the low/medium folate recombinant inbred clone USW4self#3 (referred to as USW4s#3 thereafter) to generate an F_1_ progeny. USW4s#3 is a selected self from a Minnesota advanced selection 20-20-34 (it has better than usual flowering, male fertility, and it and its progeny are known to self). Twelve of the resulting F_1_ seedlings were intermated to produce an F_2_ population named BRR3. True potato seeds from the resulting F_2_ population were soaked in GA3 at 1 g/L overnight before germination in June 2014. When plantlets reached approximately 8-cm high, they were transplanted in 8-cm square individual pots containing Sunshine Mix LA4P. All-purpose fertilizer 20-20-20 was applied at 200 mg/L once a week until senescence. Plants were watered twice a week until senescence. Vines were killed on October 31^st^, 2014, and tubers were harvested on November 11^th^. Greenhouse temperature was set at 21°C day time and 15°C night time. Supplemental light was provided for 14 hours per day from a mixture of 400 Watt high pressure sodium and 1,000 Watt metal halide lamps. While 150 seedlings were planted, only 94 individuals from the progeny produced tubers and were subsequently used for folate analysis.

Once harvested, tubers were left with skin intact, washed with cold water in a strainer, weighed, and then flash-frozen with liquid nitrogen and stored at −80°C. Frozen samples were then lyophilized in a freeze-dryer (VirTis Benchtop 4K, SP Scientific, PA) with vacuum pressure < 100 mTorr for three days. Samples were then ground to a fine powder with a Waring blender and transferred to scintillation vials for long-term storage at −80°C.

### Folate analysis

Folates were extracted by using a tri-enzyme extraction method, as previously published [18, 19]. Potato samples (100 mg) were homogenized in 15-mL Falcon tubes containing 10 mL of extraction buffer consisting of 50 mM HEPES/50 mM CHES, pH 7.85, 2% (*w/v*) sodium ascorbate and 10 mM β-mercaptoethanol and deoxygenated by flushing with nitrogen. Once homogenized, samples were boiled for 10 min and cooled immediately on ice. The homogenate was then treated with protease (≥14 units) and incubated for 2 h at 37°C, boiled again for 5 min and cooled immediately on ice. Samples were then treated with α-amylase (≥800 units) and rat plasma conjugase in excess (0.5 mL/sample), incubated for 3 h at 37°C, boiled again for 5 min and cooled immediately on ice. After centrifugation at 3,000 g for 10 min, the supernatant was transferred to a new tube. The residue was re-suspended and homogenized in 5 mL of extraction buffer, re-centrifuged for 10 min, and the supernatant was recovered. Supernatants were then combined and the samples’ volume was adjusted to 20 mL with extraction buffer. Aliquots of each sample were transferred to 1.5 mL microcentrifuge tubes, flushed with nitrogen and stored at −80°C until analysis. Controls containing all reagents, but potato samples, were used to determine the amount of residual folates in the reagents. There were no detectable folates in any of the reagents used.

Folate concentrations were measured by microbiological assay using *Lactobacillus rhamnosus*. *L*. *rhamnosus* (ATCC 7469) cultures were obtained from the American Type Culture Collection (Manassas, VA, USA). Glycerol cryoprotected cells of *L*. *rhamnosus* were prepared as described previously [18]. Assays were performed in 96-well plates (Falcon microtiter plates). Wells contained growth medium supplemented with folate standards or potato extracts, each plated in triplicate. Bacterial growth was measured at 630 nm after 18 h, 21 h and 24 h of incubation at 37°C. The 24-h reading was usually used for analysis unless saturation was reached, in which case, the 21-h reading was used. All measurements were made with a BioTek Instrument EL 311 SX microplate auto-reader (BioTekInstrument, Winooski, VT, USA), analyzed with the KCJr EIA application software (BioTekInstrument, Winooski, VT, USA) and compiled in Microsoft Excel. Folate concentrations were calculated by reference to a standard curve using 5-formyl-THF and expressed as nanograms of folate per gram of dry sample (ng·g^−1^ dry weight).

A large batch of dried potato powder from tubers of *Solanum pinnatisectum* (PI 275233) was previously prepared to be used as reference material [19]. Each batch of extractions contained 18 samples plus the reference material. Values obtained for samples were normalized to values obtained for the reference material. The average folate concentration of the reference material across all the extractions was 1,105 ± 76 ng·g^−1^ dry weight. Folate concentrations presented are normalized averages of three technical replications from single biological replication except the Fol 1.6 and USW4s#3 which are the normalized average of twelve technical replications from four biological replications. All calculations were performed with standard function settings in Microsoft Excel.

### Genomic DNA isolation

Approximately 15 mg of freeze dried tuber sample were homogenized in 600 μL CTAB extraction buffer (2% cetyltrimethyl ammonium bromide, 1.4 M NaCl, 20 mM EDTA pH 8.0, 100 mM Tris-Cl pH 8.0, 0.2% β-mercaptoethanol) in a 1.7 mL microcentrifuge tube and incubated at 65°C for 1 h with gentle mixing every 15 min. Cold chloroform (300 μL) was then added and the solution was vortexed briefly to form an emulsion. After centrifugation at 12,000 g for 5 min, the aqueous phase was transferred to a new 1.7 mL microcentrifuge tube. An equal volume of cold isopropanol was then added, the tubes were inverted several times to mix and placed on ice for 10–15 min. The resulting mix was centrifuged at 12,000 g for 10 min, the supernatant was discarded, and the pellet was washed with 300 μL of cold 70% ethanol. Samples were centrifuged for 2 min at 3,500 g. This washing step with ethanol was repeated two more times and then the pellet was re-suspended in 100 μL deionized water. Genomic DNA extracts were treated with 1 μL RNase A for 1 h at 37°C with gentle mixing every 15 min. RNase A was then inactivated by incubating samples in a water bath at 65°C for 5–10 min. Samples were then centrifuged quickly to remove bubbles and placed on ice. Samples were further cleaned by treatment with phenol and chloroform. Phenol:chloroform:isoamyl alcohol 25:24:1, pH 8.0 (100 μL) was added to the extracts in a chemical fume hood. Samples were vortexed briefly to form an emulsion. After centrifugation at 13,000 g for 15 min, the aqueous phase (~90–100 μL) was transferred to a new microcentrifuge tube. Cold chloroform (100 μL) was then added and samples were vortexed for 10 seconds. After centrifugation at 13,000 g for 15 min the aqueous phase (80–85 μL) was transferred to a new microcentrifuge tube. DNA was precipitated by addition of 3 M sodium acetate pH 5.3 (1/10 of the sample volume) and 95% ethanol (2.5 volumes) at −20°C for 2 hours or overnight. Samples were then centrifuged for 30 min at 13,000 g at 4°C. The supernatant was discarded and the pellet was washed three times with 300 μL of cold 70% ethanol as described above. Pellets were re-suspended in 30 μL deionized water and stored at -20°C.

### SNP genotyping

At least 400 ng of genomic DNA from 96 samples (94 progeny and parents) were loaded onto a 96-well microplate and desiccated in an Eppendorf Vacufuge Plus (Eppendorf North America, Hauppauge, NY) in 30 min intervals at 25°C until all samples were completely dried. Samples were then sent to GeneSeek (Neogen Corporation, Lincoln, NE) for custom SNP Profiling using the Illumina platform Infinium SolCAP 12K SNP array.

### SNP quality and filtering

The idat data file developed from 12K SolCAP array was imported to the Illumina GenomeStudio software (genotyping version) (Illumina, San Diego, CA) and analyzed for allele calling. For calling SNPs using the diploid model on the v2 SolCAP 12K array, auto-clustering was run in GenomeStudio using standard settings, followed by importing the three cluster calling files from the v1 SolCAP 8303 SNP array. All the SNPs were sorted based on GenTrain score and each of the SNP marker was manually checked for three clusters calling, i.e., AA, AB and BB type alleles (too high or low GenTrain score generally corresponds to monomorphic and bad markers, respectively). Final allelic data was exported from GenomeStudio and first filtered to remove “BAD” and “QUESTIONABLE” SNPs based on quality comments from the GeneSeek’s data summary file. After initial filtering, there were 10,120 SNPs showing amplification. Further filtering for SNPs with less than 10% missing values resulted in 6,759 high quality SNPs that were used for SNP-trait association study.

### Population structure and marker properties

Allelic data was first used to survey any kind of internal structure, if present in the population. Factorial analysis (dissimilarity) and neighbor joining (unweighted neighbor joining) was performed using all the polymorphic makers in Darwin (http://darwin.cirad.fr/darwin) [31]. Polymorphic information content (PIC), heterozygosity (Het), diversity (Div), and minor allele frequency (MAF) for significant markers were calculated using JMP Genomics 8 (JMP, A Business Unit of SAS Cary, NC).

### Marker-trait association analysis

SNP-trait association analysis was performed in JMP Genomics 8 (JMP, A Business Unit of SAS Cary, NC) using two different approaches: STA and SSTA. Both STA and SSTA analyses are the best fit for analyzing bi-allelic markers for marker-trait association. In brief, folate data was log transformed to fit a more continuous distribution. The annotated chromosomal locations provided from the SNP array was used for the analyses. Datasets were transformed into SAS format “sas7bdat” and uploaded into JMP Genomics. Both functions were run at default parameters for folate content as a continuous trait distribution, with non-delimited genotypes, Benjamini and Hochberg correction (FDR) and Fisher based p-value adjustment. SSTA treats genotypes as categorical variables and uses an ANOVA function for genotypes. STA uses SAS PROC MIXED approach to find associations between markers and the continuous traits.

## Results and discussion

### Folate content

The F_2_ segregating population was developed by crossing a high folate wild diploid *S*. *boliviense* parent (Fol 1.6) with a low folate diploid *S*. *tuberosum* parent (USW4s#3), and eventually intermating F_1_ individuals. F_2_ individuals were grown throughout the summer and fall in a greenhouse, but many did not tuberize. The lack of tuberization from one third of the interspecific population is mainly attributed to alleles from *S*. *bolivense*, which usually produces tubers under short day conditions. Percent tuberization (62%) of BRR3 population is within the range of previously reported haploid-wild species hybrid [32]. Tubers from 94 F_2_ individuals were used for folate analysis and SNP genotyping.

**Fig 1:**
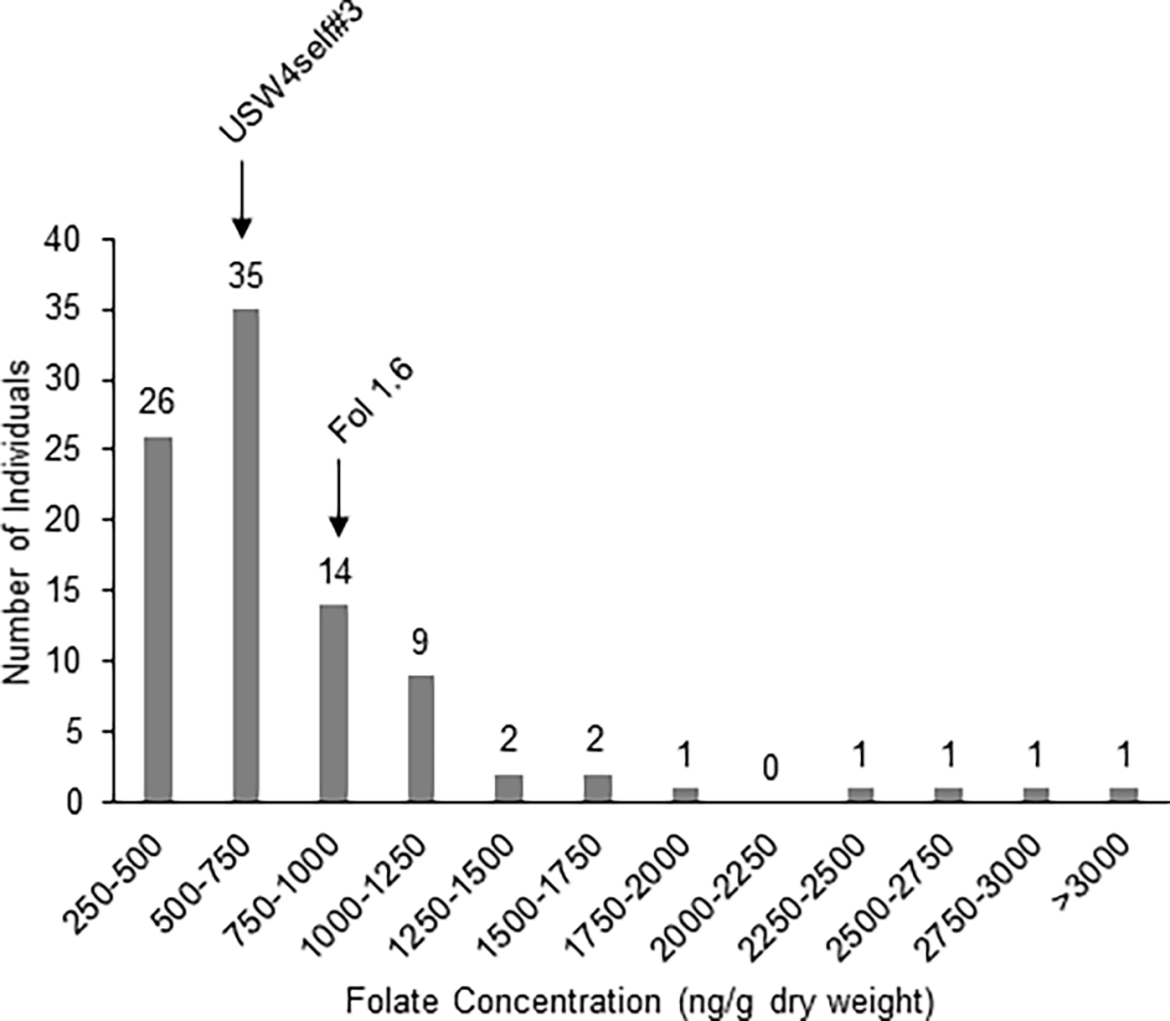
Distribution of folate concentrations in the BRR3 F_2_ progeny. Histogram represents number of individuals within folate concentration ranges. Folate concentration of parents USW4s#3 and Fol 1.6 were 639 ± 71 and 950 ± 130 ng g^-1^ dry weight, respectively.

Folate content in the F_2_ BRR3 population ranged from 304 ± 16 to 2,952 ± 276 ng g^−1^ dry weight, representing a 10-fold difference between the lowest and highest folate concentrations. The majority of individuals tested (52% of all progeny) had folate concentrations between 500 and 1,000 ng g^−1^ dry weight (Fig 1). Approximately 40% of individuals showed folate concentrations below 500 or between 1,000 and 1,500 ng g^−1^ dry weight. The remaining ~8% had folate concentrations above 1,500 ng g^−1^ dry weight, with three individuals between 1,500 and 2,000 ng g^−1^ dry weight and four above 2,000 ng g^−1^ dry weight (Fig 1). The parents Fol 1.6 and USW4s#3 had folate concentrations of 950 ± 130 and 639 ± 71 ng g^−1^ dry weight, respectively. It is clear from these results that the folate content trait shows transgression or transgressive segregation. The presence of extreme or transgressive phenotypes in segregating hybrid populations is common as previously reported [33]. Transgression can be caused by the action of complementary genes, overdominance and epistasis [33]. Note that values presented for the female parent USW4s#3 and the seedlings were from the 2014 growing season, while values presented for Fol 1.6 were from tubers harvested in 2015. Fol 1.6 was chosen as a parent in this cross because it showed tuber folate levels above 1,600 ng g^−1^ dry weight in two previous harvests in 2011 and 2012. The lower values of Fol 1.6 presented here can probably be attributed to the particular growing conditions. The trait reliability estimate (broad sense heritability) of folate content from a *S*. *microdontom* population [34] is 0.67 [unpublished, Goyer et al., *In Prep*], suggesting that the folate trait is highly heritable and that high folate plants tend to consistently have higher folate content than low folate plants.

### Selection of SNP markers

A total of 6,759 high-quality SNPs (4,174 polymorphic and 2,585 monomorphic) were selected for mapping after very stringent screening of the 12K SNP array using the GenTrain score in Genome Studio. Sample BRR80 was removed from further analysis as it behaved as an off type for the majority of monomorphic markers. All the SNPs selected showed less than 10% “No call rate”.

SNP genotyping showed that the USW4s#3 parent is moderately heterozygous (34.8%), and that the *S*. *boliviense* parent Fol 1.6 is less heterozygous (5.3%) (S1 Table). Our initial assumption was that both parents used in this study were highly homozygous and hence we developed an F_2_ segregation population by intermating twelve F_1_ plants. The presence of moderate levels of heterozygosity in the parents could explain the unexpected segregation distortion among the F_2_ population, which can consequently not be considered a true biparental population. Hence, the conventional QTL mapping could not prove effective in the present study. Instead, in such random mated, heterogeneous population, the most effective strategy to identify marker-trait association is association mapping. Association mapping identifies QTL by calculating marker-trait associations that could be attributed to linkage disequilibrium between markers and functional polymorphisms in diverse individuals [35]. It is often more rapid and cost-effective than traditional linkage mapping. Linkage and association mapping are complementary approaches and are more similar than often assumed [36, 37].

### Population structure

**Fig 2:**
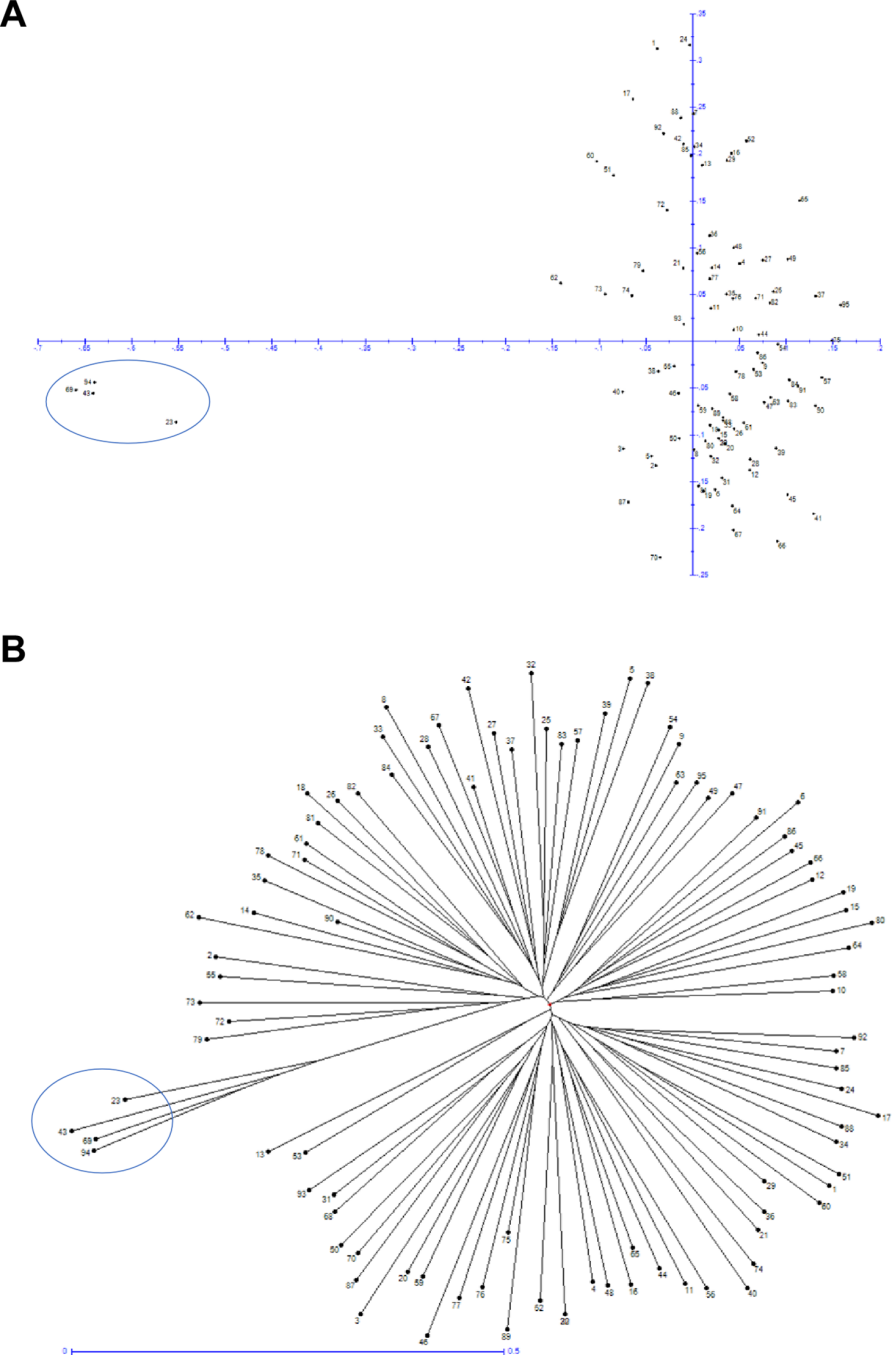
Population structure of the F_2_ individuals used in this study. (A) Factorial analysis of the 95 samples showing the absence of any subgroups. Four of the samples [BRR13 (#23), BRR27 (#43), BRR6 (#69) and Fol 1.6 (#94)] can be clearly seen as outliers in factorial analysis (Axes ½) (circled). (B) Neighbor Joining (NJ) tree showing the four outliers in the population used in the present study.

Population structure is one of the major constraints that might skew the results of association studies [38]. As association mapping evaluates whether specific alleles within a population are linked with specific phenotypes more frequently than expected [39], the presence of population structure within the samples could lead to spurious linkages/associations [40]. Population structure of the F_2_ individuals was surveyed to identify any accidental selfing and unrelated outcrossing individuals. All the individuals were scanned using factorial analysis (based on dissimilarity with number of axes set at 5) and neighbor joining (based on unweighted pairwise). Both these analyses suggest the absence of any potential subgroups (outcrossing groups). However, four samples (BRR13, BRR27, BRR6 and Fol 1.6) clustered together to form a separate group (Fig 2, numbers 23, 43, 69, and 94, respectively, and percent heterozygosity in S1 Table). Percent heterozygosity analysis of these individuals revealed that these were the least heterozygous individuals in the population (S1 Table). The average heterozygosity in the population is 19.9% while these individuals have an average of 6.3% heterozygosity, which is almost equal to the Fol 1.6 parent (5.3%). The heterozygosity values suggest that these individuals are the product of accidental selfing.

### Marker-trait association

Although larger population sizes increase the power of marker-trait associations, previous studies have used population sizes similar to the one used in this study [41, 42]. Association mapping analyses were run with and without the aforementioned outliers but the results remained unaffected by the presence or absence of these outliers. Therefore, they were included in the final analyses. Two different marker trait association approaches available in JMP Genomics, SSTA and STA, were used in this study. Both these methods have been designed to handle large-scale bi-allelic genetic data with known locations on the chromosomes to test association with quantitative as well as qualitative phenotypic traits. SSTA can handle complex survey designs to depict marker-trait association by testing a single SNP at a time. It performs two types of analysis, SNP markers based ANOVA testing and REGRESSION analysis for qualitative covariates. STA performs marker-trait association analysis by using SAS PROC MIXED model for continuous traits.

SSTA and STA analyses using genotype test identified 489 and 43 significant markers, respectively, with -log10(p) ≥ 1.3 (default parameter in JMP Genomics) (Table 1, S2 and S3 Tables, S1 Fig). These results show that the mixed model approach is more stringent and hence reduces the number of false positives. The mixed model approach is more powerful as it can handle the internal population structure by accommodating the phenotypic covariance that could be due to genetic relatedness [43]. As STA considers neighboring markers for association while SSTA surveys for single marker trait association without any influence of linkage, it is expected to see more marker associations from the later approach.

**Table 1:** Total number of significant markers [-log10(p) ≥ 1.3] in each chromosome resulting from two different association mapping approaches, survey SNP-trait association (SSTA) and SNP-trait association (STA) (genotype test).

Chromosome	SSTA	STA
Chr. 0	16	0
Chr. 1	71	0
Chr. 2	43	1
Chr. 3	152	0
Chr. 4	31	7
Chr. 5	28	0
Chr. 6	50	35
Chr. 7	60	0
Chr. 8	4	0
Chr. 9	15	0
Chr. 10	5	0
Chr. 11	1	0
Chr. 12	13	0
Total	489	43

SNP, single nucleotide polymorphism.

**Fig 3:**
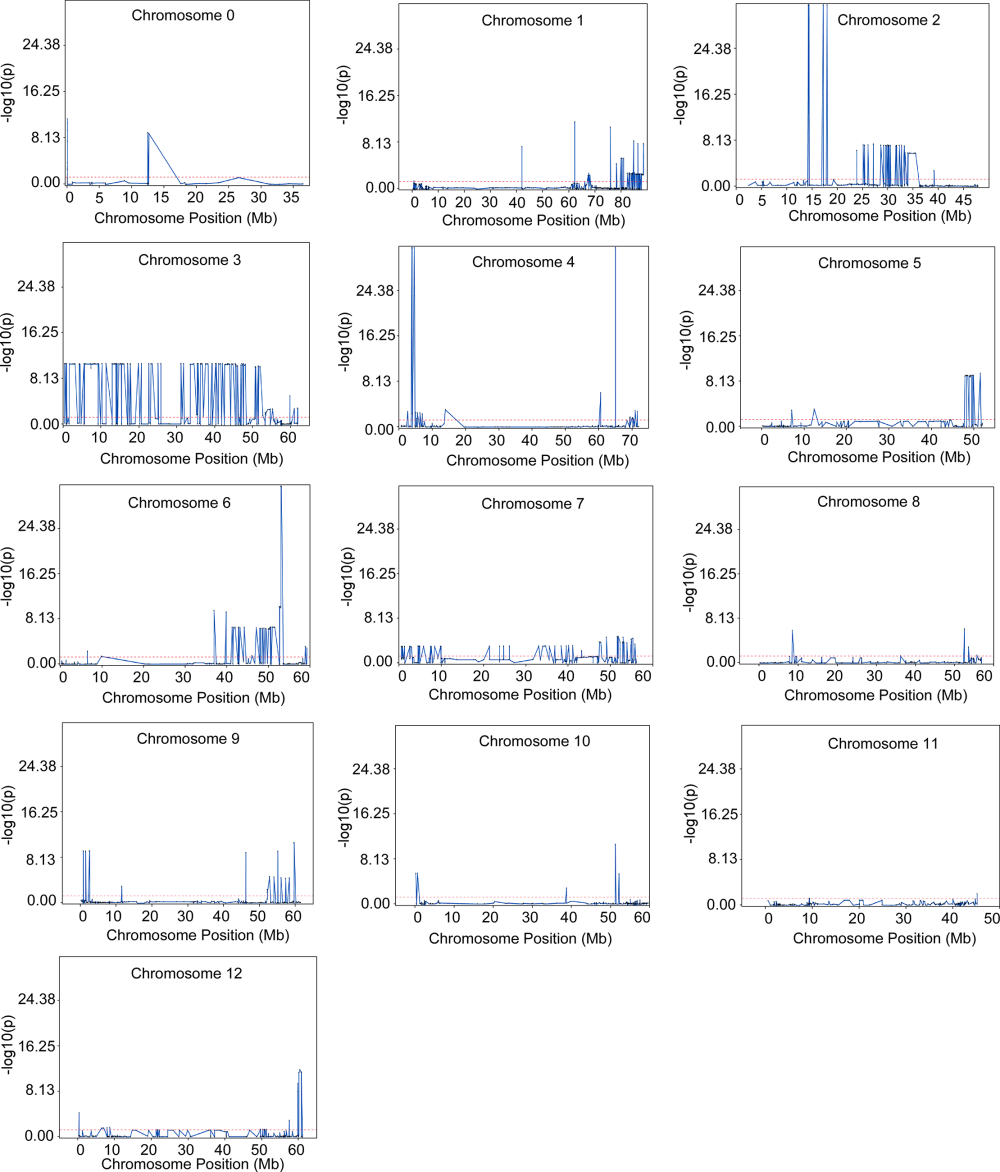
Overlay plot showing markers on each of twelve chromosomes that displayed significant association with folate content using survey SNP-trait association (SSTA) analysis. A -log10(p) ≥ 1.3 cutoff was set for significance (default parameter in JMP Genomics). SNP, single nucleotide polymorphism.

**Fig 4:**
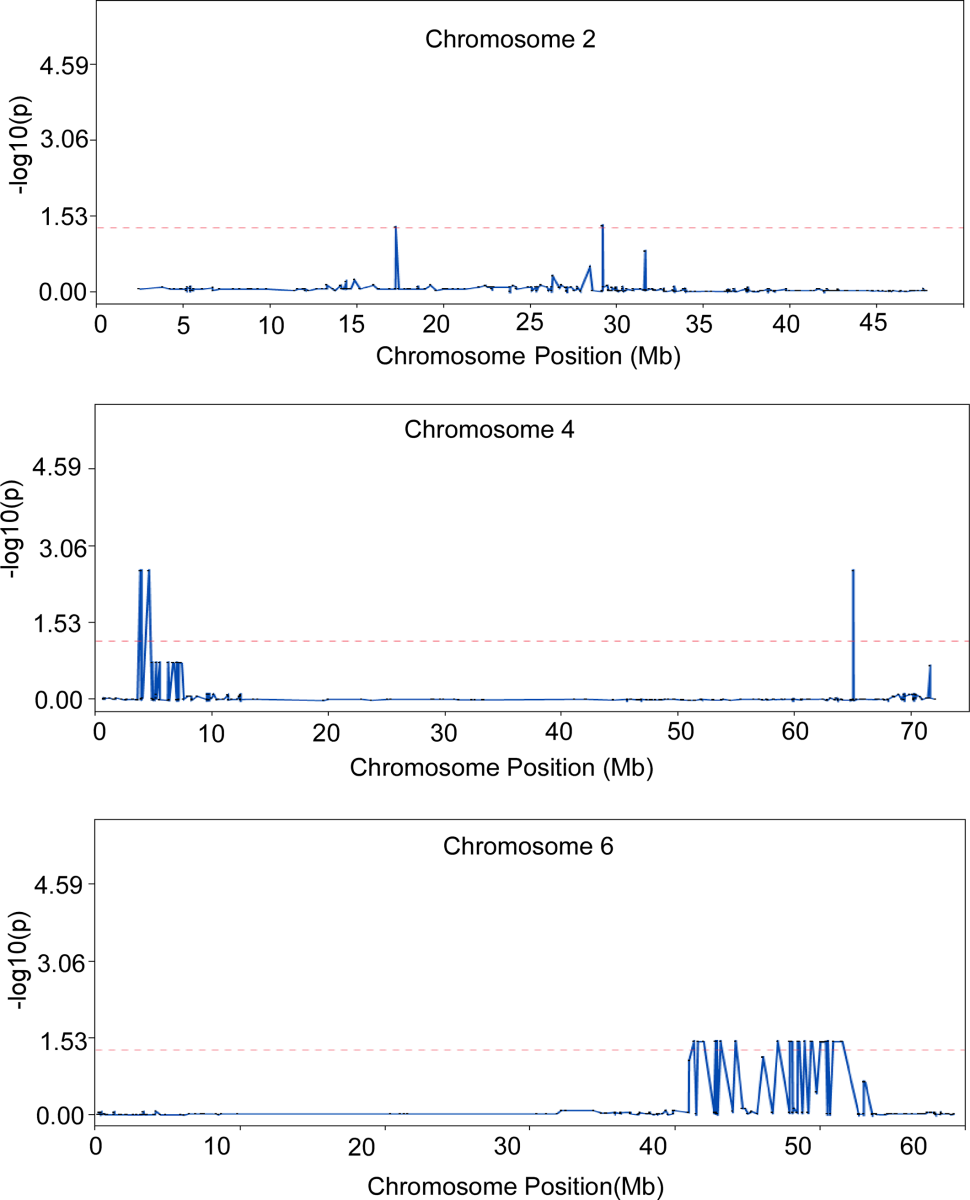
Overlay plot showing markers on chromosomes 2, 4, and 6 that displayed significant association with the folate trait using SNP-trait association (STA) analysis. A -log10(p) ≥ 1.3 cutoff was set for significance (default parameter in JMP Genomics). SNP, single nucleotide polymorphism.

SSTA identified 489 significant markers located on all twelve chromosomes, with chromosome 3 containing the highest number of markers (Table 1, Fig 3, S2 Table). The large number of SNPs identified from SSTA can be attributed to the fact that this approach uses linear regression. Furthermore, the large number of significant SNPs may also point to the overall homozygosity of Fol 1.6 parent (S1 Table) and low level of recombination events in chromosome 3 (calculated average recombination frequency on 152 markers is 20.89). STA analysis identified 43 significant markers that were located on three chromosomes only, chromosomes 2 (one marker), 4 (seven markers), and 6 (35 markers) (Table 1, Fig 4, S3 Table). Chromosomes 4 and 6 showed recombination hotspots and the average recombination frequency was 37.21 and 40.34, respectively. The significant markers on chromosomes 2, 4 and 6 identified by STA analysis could explain an average of 15%, 24% and 16% variance, respectively (S3 Table).

Of particular interest are significant SNPs that were identified by both SSTA and STA methods. There were 50 and 35 significant markers on chromosome 6 identified by SSTA and STA analyses, respectively, out of which 35 were common in both analyses. Similarly, STA identified seven markers on chromosome 4, whereas SSTA identified 31 markers on the same chromosome, out of which seven were common in both analyses. This set of markers showed an average of 0.29 PIC, 0.43 Het, 0.36 Div, and 0.24% MAF (Table 2). Similar values have been reported earlier in a collection of Andigenum group used for genetic diversity and association mapping with SolCap array SNP markers [30]. MAF determines the accuracy of marker trait association. It generally ranges between 0.01 and 0.50 and the power to detect significant markers increases as MAF increases [44]. MAF under 5–10% can artificially increase the association score, thereby resulting in spurious associations. Other genetic indices like PIC, Het and Div could be considered within range for a mapping population with narrow genetic base.

**Table 2:** Polymorphic information content (PIC), heterozygosity (Het), diversity (Div), minor allele frequency (MAF), and % variance of 42 most significant markers identified in the present study.

Chromosome	Position	Lab_code	SolCap ID	PIC	Het	Div.	MAF	% variance
Chr. 6	51484815	FolSNP195	solcap_snp_c1_13135	0.30	0.44	0.37	0.24	0.169958708
Chr. 6	48433233	FolSNP341	solcap_snp_c1_15368	0.29	0.40	0.35	0.22	0.165213348
Chr. 6	48416539	FolSNP342	solcap_snp_c1_15371	0.29	0.40	0.35	0.22	0.165213348
Chr. 6	48416425	FolSNP343	solcap_snp_c1_15372	0.28	0.39	0.34	0.22	0.167203989
Chr. 6	50219978	FolSNP474	solcap_snp_c1_2060	0.29	0.42	0.36	0.23	0.161668231
Chr. 6	47847667	FolSNP1366	solcap_snp_c2_16778	0.29	0.41	0.35	0.23	0.167968014
Chr. 6	48020360	FolSNP1367	solcap_snp_c2_16780	0.29	0.41	0.35	0.23	0.167968014
Chr. 6	48874844	FolSNP2172	solcap_snp_c2_31139	0.28	0.40	0.34	0.22	0.165669449
Chr. 6	48503815	FolSNP2174	solcap_snp_c2_31188	0.28	0.39	0.34	0.22	0.165714421
Chr. 6	48529862	FolSNP2175	solcap_snp_c2_31214	0.28	0.38	0.34	0.21	0.166863779
Chr. 6	49958510	FolSNP2354	solcap_snp_c2_33863	0.29	0.42	0.36	0.23	0.161668231
Chr. 6	49407305	FolSNP2470	solcap_snp_c2_35889	0.28	0.40	0.34	0.22	0.162793288
Chr. 6	49305939	FolSNP2471	solcap_snp_c2_35897	0.28	0.40	0.34	0.22	0.162793288
Chr. 6	43114810	FolSNP2597	solcap_snp_c2_37770	0.28	0.40	0.34	0.22	0.170162409
Chr. 6	42803660	FolSNP2900	solcap_snp_c2_43116	0.28	0.39	0.34	0.22	0.171076302
Chr. 6	42830329	FolSNP2902	solcap_snp_c2_43124	0.28	0.39	0.34	0.22	0.17418471
Chr. 6	47053482	FolSNP3129	solcap_snp_c2_46172	0.29	0.40	0.35	0.22	0.169378479
Chr. 6	47027778	FolSNP3130	solcap_snp_c2_46184	0.29	0.41	0.35	0.23	0.169486686
Chr. 6	41241077	FolSNP3300	solcap_snp_c2_48886	0.28	0.38	0.33	0.21	0.173980285
Chr. 6	41926673	FolSNP3322	solcap_snp_c2_49048	0.28	0.40	0.34	0.22	0.170162409
Chr. 6	41927092	FolSNP3323	solcap_snp_c2_49052	0.28	0.40	0.34	0.22	0.170162409
Chr. 6	41927126	FolSNP3324	solcap_snp_c2_49053	0.28	0.39	0.34	0.22	0.17418471
Chr. 6	48462817	FolSNP3547	solcap_snp_c2_52575	0.29	0.40	0.35	0.22	0.165213348
Chr. 6	48405097	FolSNP3548	solcap_snp_c2_52583	0.29	0.40	0.35	0.22	0.165213348
Chr. 6	44119412	FolSNP3580	solcap_snp_c2_53053	0.28	0.38	0.33	0.21	0.173980285
Chr. 6	41520176	FolSNP3639	solcap_snp_c2_54029	0.28	0.38	0.33	0.21	0.173980285
Chr. 6	51365568	FolSNP3772	solcap_snp_c2_56141	0.30	0.44	0.37	0.24	0.170321941
Chr. 6	42746310	FolSNP3824	solcap_snp_c2_57014	0.28	0.40	0.34	0.22	0.170162409
Chr. 6	42759644	FolSNP3825	solcap_snp_c2_57017	0.28	0.40	0.34	0.22	0.170162409
Chr. 6	43086245	FolSNP3859	solcap_snp_c2_57412	0.28	0.39	0.34	0.22	0.171076302
Chr. 6	50345098	FolSNP3880	solcap_snp_c2_5772	0.29	0.41	0.35	0.23	0.16865084
Chr. 6	50470736	FolSNP3898	solcap_snp_c2_5812	0.29	0.41	0.35	0.23	0.16865084
Chr. 6	50088968	FolSNP3920	solcap_snp_c2_5836	0.30	0.43	0.36	0.24	0.161363089
Chr. 6	50851628	FolSNP3928	solcap_snp_c2_5858	0.30	0.43	0.36	0.24	0.171426966
Chr. 6	50109162	FolSNP3929	solcap_snp_c2_5869	0.29	0.42	0.36	0.23	0.161668231
Chr. 4	64973061	FolSNP775	solcap_snp_c1_6749	0.30	0.46	0.37	0.24	0.249706513
Chr. 4	3905183	FolSNP953	solcap_snp_c1_9546	0.33	0.58	0.42	0.30	0.247786574
Chr. 4	4606550	FolSNP1672	solcap_snp_c2_21934	0.33	0.58	0.42	0.30	0.247786574
Chr. 4	4595286	FolSNP1673	solcap_snp_c2_21936	0.33	0.58	0.42	0.30	0.251816755
Chr. 4	4567755	FolSNP1674	solcap_snp_c2_21946	0.33	0.58	0.42	0.30	0.247786574
Chr. 4	3816447	FolSNP2206	solcap_snp_c2_31688	0.33	0.58	0.42	0.30	0.247786574
Chr. 4	3924918	FolSNP2210	solcap_snp_c2_31732	0.33	0.58	0.42	0.30	0.247786574

These common significant SNPs span a region of 10.24 Mb in chromosome 6. In chromosome 4, the common SNPs are located in two hotspots that are 60 Mb apart, one of which spans 0.7 Mb and consists of six SNPs, whereas the second spot consisted of only one SNP marker. Hence, these 42 SNPs located on chromosome 6 and 4 (Table 2) could be considered as best candidate markers for high folate content.

One possible weakness of SNP as a marker is ascertainment bias [22]. The use of a biased set of pre-ascertained SNPs designed from *S*. *tuberosum* genome are more likely to address only the common alleles rather than the rare alleles from *S*. *bolivense*. This could result in loss of differential real folate alleles from *S*. *bolivense*. *S*. *bolivense* population shows segregation for folate trait, but the underlying alleles, even if segregating, are less likely to be marked up by the SNPs derived from *S*. *tuberosum*.

### Location of SNPs and folate genes

Folate biosynthesis is a well characterized pathway in plants [45]. It involves ten enzymatic steps that are catalyzed by nine proteins (S4 Table). Three enzymes that may be involved in salvage and/or homeostasis of folate have also been characterized [46–49] (S4 Table). Searches for potato homologs of Arabidopsis and tomato genes revealed 16 folate-related genes in the potato reference genome that are located on different chromosomes (S4 Table). Comparison of genomic location of these genes and that of the 497 SNPs identified by SSTA and STA analyses showed that one significant SNP was located within the 5-FCL gene, and 17 additional SNPs were located in close proximity (<0.1 Mb) to folate metabolism genes (Table 3). We hypothesize that there are two major QTLs located in chromosome 4 and 6, and additional minor QTLs in other chromosomes that contribute to folate biosynthesis.

**Table 3:** Location of folate metabolism-related genes on potato chromosomes and closest significant SNPs.

Folate gene	Chromosome	Closest SNPs (SSTA, STA, or both)	Distance from folate gene (Mb)
DHFR	Chr. 1	solcap_snp_c2_49910 (SSTA)	<0.1
DHNTP-PPase	Chr. 3	solcap_snp_c2_50637 (SSTA)	<0.6
**5-FCL**	Chr. 3	solcap_snp_c1_5799 (SSTA)	<0.0
DHNA	Chr. 4	solcap_snp_c2_21636 (SSTA) solcap_snp_c2_21934 (both)	<2.6 <6.8
**ADCS**	Chr. 4	solcap_snp_c2_21636 (SSTA)	<17
HPPK/DHPS	Chr. 5	solcap_snp_c2_55452 (SSTA)	<6.3
**FPGS**	Chr. 5	solcap_snp_c2_10287 (SSTA)	<0.1
GCHI	Chr. 6	solcap_snp_c2_9233 (SSTA) solcap_snp_c2_5858 (STA) solcap_snp_c1_13135 (both)	<0.7 <7.4 <6.8
**DHFS**	Chr. 6	solcap_snp_c2_42355 (SSTA) solcap_snp_c2_48886 (both)	<1.3 <3.1
GGH1	Chr. 7	solcap_snp_c2_42761 (SSTA)	<0.3
**GGH3**	Chr. 7	solcap_snp_c2_42761 (SSTA)	<0.3
DHNA	Chr. 10	solcap_snp_c2_51076 (SSTA)	<5.9
**GGH2**	Chr. 10	solcap_snp_c1_329 (SSTA)	<2.0
ADCL	Chr. 11	solcap_snp_c2_34204 (SSTA)	<2.5
UDP-Glu-pABA glucosyltransferase	Chr. 12	solcap_snp_c2_5333 (SSTA)	<0.4

SNP, single nucleotide polymorphism; SSTA, survey SNP-trait association; STA, SNP-trait association; DHFR, dihydrofolate reductase; DHNTP-PPase, dihydroneopterin triphosphate diphosphatase; 5-FCL, 5-formyltetrahydrofolate cycloligase; DHNA, dihydroneopterin aldolase; ADCS, aminodeoxychorismate synthase; HPPK/DHPS, 6-hydroxymethyldihydropterin pyrophosphokinase/ dihydropteroate synthase; FPGS, folylpolyglutamate synthase; GCHI, GTP cyclohydrolase I; DHFS, dihydrofolate synthase; GGH, γ-glutamyl hydrolase; ADCL, aminoedoxychorismate lyase; UDP-Glu-pABA glucosyltransferase, UDP-glucose-*p*-aminobenzoate glucosyltransferase.

## Conclusions

In this study, a diploid segregating population was developed and genotyped to find significant associations between SNPs and folate content. A total of 497 SNPs were identified with potential associations with folate content by using two analytical methods. A set of 42 identical SNPs located on chromosome 6 and 4 was identified by both methods. Additional significant SNPs were located within or in close proximity to folate metabolism-related genes. Considering high reliability of folate trait with presence of high frequency of minor alleles, the set of markers identified in this study could facilitate the systematic screening of high folate germplasm and the introgression of the high-folate trait into new potato varieties. Because folate analyses are tedious and materials used in this study cannot really be evaluated in the field, folate was quantified from a single tuber harvest. Better replication, validation, and further tests in a field-ready background are next steps to this preliminary work. Moreover, there may be variation between genotypes in terms of folate retention after cooking. It will be important to evaluate and select germplasm that maintains high folate content after cooking.

## Supporting information

S1 Fig Number of significant markers (-log(p) ≥ 1.3) on each of the twelve potato chromosomes. (A) Survey SNP-trait association (SSTA) analysis. (B) SNP-trait association (STA) analysis. SNP, single nucleotide polymorphism.
(TIF)

S1 Table Percent heterozygosity of the population used in association mapping study. (DOCX)

S2 Table List of significant SNPs (-log(p) ≥ 1.3) identified by SSTA analysis. In red are markers that displayed high p-values but were included in the study because they are common significant markers in both SSTA and STA. SSTA, survey SNP-trait association; STA, SNP-trait association; SNP, single nucleotide polymorphism.
(XLSX)

S3 Table List of significant SNPs (-log(p) ≥ 1.3) identified by STA analysis. STA, SNP-trait association; SNP, single nucleotide polymorphism.
(XLSX)

S4 Table Folate metabolism-related genes in *Arabidopsis*, tomato, and potato. Biosynthesis genes are highlighted in blue while salvage and homeostasis genes are highlighted in green.
(DOCX)
