# Supplementary figures and images for: Single Nucleotide Polymorphism (SNP) markers associated with high folate content in wild potato species
Source: PLoS One. 2018 Feb 23;13(2):e0193415. doi: 10.1371/journal.pone.0193415 (PMC5825101; doi:10.1371/journal.pone.0193415)

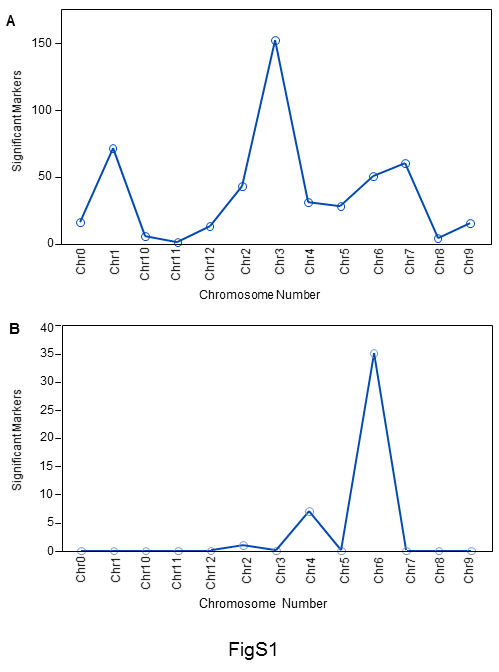

Supplement: S1 Fig — (A) Survey SNP-trait association (SSTA) analysis. (B) SNP-trait association (STA) analysis. SNP, single nucleotide polymorphism. (TIF) [file pone.0193415.s001.tif]
