# Supplementary material for: Single Nucleotide Polymorphism (SNP) markers associated with high folate content in wild potato species
Source: PLoS One. 2018 Feb 23;13(2):e0193415. doi: 10.1371/journal.pone.0193415 (PMC5825101; doi:10.1371/journal.pone.0193415)
Supplement: S1 Table — (DOCX) [file pone.0193415.s002.docx]

**S1 Table. Percent heterozygosity of the population used in association mapping study.**

| **Sample** | **--** | **AA** | **AC** | **AG** | **AT** | **CC** | **CG** | **GC** | **GG** | **TA** | **TC** | **TG** | **TT** | **%Het** |
| --- | --- | --- | --- | --- | --- | --- | --- | --- | --- | --- | --- | --- | --- | --- |
| Fol1.6 | 404 | 2028 | 50 | 217 | 10 | 2584 | 4 | 5 | 2670 | 14 | 178 | 40 | 1916 | 5.3 |
| USW4S3 | 210 | 1442 | 356 | 1341 | 55 | 1821 | 37 | 28 | 1842 | 68 | 1274 | 292 | 1354 | 34.8 |
| BRR001 | 380 | 1830 | 157 | 648 | 27 | 2310 | 15 | 12 | 2317 | 37 | 561 | 138 | 1688 | 16.4 |
| BRR002 | 322 | 1653 | 238 | 891 | 37 | 2139 | 22 | 19 | 2186 | 43 | 817 | 196 | 1557 | 23.1 |
| BRR003 | 289 | 1723 | 224 | 822 | 37 | 2153 | 27 | 20 | 2206 | 40 | 797 | 166 | 1616 | 21.7 |
| BRR004 | 337 | 1740 | 183 | 722 | 27 | 2242 | 18 | 14 | 2285 | 31 | 690 | 172 | 1659 | 19.0 |
| BRR006 | 561 | 1958 | 54 | 227 | 14 | 2554 | 7 | 3 | 2637 | 18 | 194 | 37 | 1856 | 5.8 |
| BRR007 | 337 | 1745 | 189 | 724 | 31 | 2251 | 20 | 12 | 2309 | 29 | 676 | 140 | 1657 | 18.6 |
| BRR008 | 328 | 1641 | 234 | 894 | 31 | 2157 | 23 | 24 | 2187 | 40 | 805 | 199 | 1557 | 23.0 |
| BRR012 | 306 | 1794 | 185 | 643 | 26 | 2305 | 18 | 10 | 2363 | 28 | 605 | 126 | 1711 | 16.7 |
| BRR013 | 831 | 1059 | 43 | 214 | 14 | 1617 | 6 | 1 | 1735 | 13 | 169 | 43 | 1014 | 8.4 |
| BRR014 | 297 | 1731 | 206 | 795 | 29 | 2169 | 23 | 18 | 2233 | 37 | 776 | 178 | 1628 | 21.0 |
| BRR015 | 346 | 1725 | 211 | 791 | 28 | 2183 | 28 | 18 | 2219 | 41 | 736 | 178 | 1616 | 20.8 |
| BRR016 | 292 | 1589 | 238 | 979 | 39 | 2126 | 33 | 15 | 2126 | 47 | 899 | 225 | 1512 | 25.2 |
| BRR019 | 403 | 1835 | 157 | 579 | 24 | 2288 | 16 | 11 | 2373 | 36 | 612 | 127 | 1659 | 16.1 |
| BRR022 | 404 | 1788 | 166 | 639 | 25 | 2313 | 17 | 19 | 2354 | 32 | 585 | 108 | 1670 | 16.4 |
| BRR024 | 352 | 1746 | 184 | 704 | 33 | 2221 | 23 | 17 | 2294 | 32 | 672 | 167 | 1675 | 18.8 |
| BRR027 | 490 | 2001 | 51 | 207 | 12 | 2556 | 7 | 6 | 2646 | 17 | 199 | 37 | 1891 | 5.6 |
| BRR032 | 319 | 1689 | 225 | 915 | 26 | 2079 | 25 | 16 | 2103 | 47 | 885 | 219 | 1572 | 24.1 |
| BRR033 | 366 | 1821 | 168 | 636 | 25 | 2251 | 11 | 13 | 2328 | 26 | 641 | 140 | 1694 | 17.0 |
| BRR037 | 330 | 1707 | 225 | 831 | 36 | 2163 | 25 | 19 | 2202 | 37 | 771 | 181 | 1593 | 21.7 |
| BRR038 | 336 | 1735 | 181 | 717 | 23 | 2229 | 23 | 19 | 2298 | 43 | 695 | 171 | 1650 | 19.1 |
| BRR039 | 290 | 1633 | 249 | 943 | 31 | 2123 | 26 | 24 | 2148 | 39 | 849 | 198 | 1567 | 24.0 |
| BRR040 | 345 | 1827 | 157 | 588 | 22 | 2308 | 18 | 12 | 2378 | 30 | 560 | 129 | 1746 | 15.5 |
| BRR041 | 320 | 1661 | 207 | 846 | 35 | 2206 | 23 | 22 | 2213 | 40 | 754 | 185 | 1608 | 21.6 |
| BRR042 | 325 | 1737 | 201 | 805 | 30 | 2141 | 24 | 24 | 2234 | 36 | 795 | 182 | 1586 | 21.4 |
| BRR043 | 319 | 1678 | 244 | 891 | 37 | 2108 | 19 | 18 | 2143 | 47 | 873 | 211 | 1532 | 23.9 |
| BRR044 | 359 | 1807 | 152 | 679 | 29 | 2276 | 16 | 17 | 2305 | 28 | 607 | 154 | 1691 | 17.2 |
| BRR045 | 342 | 1848 | 169 | 598 | 25 | 2266 | 19 | 11 | 2371 | 30 | 587 | 115 | 1739 | 15.9 |
| BRR047 | 264 | 1611 | 244 | 966 | 45 | 2077 | 31 | 19 | 2150 | 42 | 945 | 220 | 1506 | 25.5 |
| BRR048 | 337 | 1710 | 191 | 769 | 28 | 2225 | 24 | 16 | 2281 | 40 | 714 | 166 | 1619 | 19.9 |
| BRR049 | 308 | 1698 | 221 | 847 | 24 | 2175 | 20 | 21 | 2216 | 44 | 787 | 174 | 1585 | 21.8 |
| BRR050 | 314 | 1864 | 147 | 602 | 24 | 2312 | 16 | 16 | 2366 | 23 | 575 | 137 | 1724 | 15.7 |
| BRR051 | 328 | 1715 | 224 | 807 | 33 | 2207 | 20 | 12 | 2248 | 30 | 693 | 169 | 1634 | 20.3 |
| BRR052 | 359 | 1856 | 119 | 542 | 25 | 2391 | 13 | 10 | 2450 | 28 | 462 | 94 | 1771 | 13.2 |
| BRR053 | 945 | 1416 | 255 | 1009 | 40 | 1894 | 29 | 20 | 1935 | 43 | 943 | 219 | 1372 | 27.9 |
| BRR054 | 326 | 1738 | 187 | 775 | 30 | 2224 | 17 | 15 | 2250 | 40 | 706 | 151 | 1661 | 19.6 |
| BRR055 | 316 | 1650 | 231 | 896 | 35 | 2153 | 33 | 19 | 2178 | 41 | 854 | 178 | 1536 | 23.3 |
| BRR056 | 323 | 1662 | 224 | 889 | 36 | 2178 | 18 | 17 | 2181 | 36 | 787 | 197 | 1572 | 22.5 |
| BRR057 | 344 | 1720 | 178 | 750 | 21 | 2240 | 17 | 19 | 2287 | 38 | 698 | 152 | 1656 | 19.2 |
| BRR058 | 311 | 1745 | 201 | 801 | 31 | 2155 | 24 | 14 | 2203 | 40 | 781 | 192 | 1622 | 21.2 |
| BRR060 | 356 | 1745 | 184 | 720 | 30 | 2268 | 19 | 19 | 2308 | 34 | 643 | 140 | 1654 | 18.3 |
| BRR061 | 349 | 1711 | 216 | 816 | 23 | 2194 | 22 | 12 | 2216 | 35 | 726 | 184 | 1616 | 20.8 |
| BRR062 | 329 | 1791 | 168 | 714 | 26 | 2273 | 23 | 13 | 2307 | 33 | 667 | 134 | 1642 | 18.2 |
| BRR063 | 355 | 1857 | 142 | 594 | 21 | 2308 | 18 | 15 | 2356 | 30 | 569 | 141 | 1714 | 15.7 |
| BRR066 | 341 | 1791 | 183 | 634 | 27 | 2290 | 26 | 16 | 2347 | 29 | 580 | 149 | 1707 | 16.8 |
| BRR068 | 861 | 1417 | 289 | 1135 | 49 | 1829 | 32 | 26 | 1868 | 44 | 1034 | 214 | 1322 | 30.5 |
| BRR069 | 289 | 1722 | 194 | 817 | 33 | 2233 | 29 | 15 | 2239 | 33 | 687 | 153 | 1676 | 19.9 |
| BRR070 | 357 | 1781 | 173 | 684 | 31 | 2287 | 13 | 7 | 2330 | 25 | 604 | 136 | 1692 | 17.1 |
| BRR071 | 350 | 1856 | 140 | 585 | 24 | 2356 | 20 | 14 | 2378 | 27 | 543 | 105 | 1722 | 14.9 |
| BRR073 | 352 | 1805 | 176 | 696 | 31 | 2244 | 21 | 16 | 2285 | 32 | 672 | 152 | 1638 | 18.4 |
| BRR078 | 328 | 1755 | 182 | 727 | 20 | 2260 | 21 | 13 | 2328 | 33 | 661 | 142 | 1650 | 18.4 |
| BRR080 | 906 | 890 | 471 | 2074 | 75 | 1178 | 51 | 32 | 1160 | 93 | 1819 | 433 | 938 | 54.8 |
| BRR081 | 336 | 1611 | 239 | 895 | 36 | 2140 | 30 | 24 | 2184 | 39 | 857 | 199 | 1530 | 23.7 |
| BRR083 | 341 | 1656 | 212 | 833 | 25 | 2210 | 21 | 17 | 2257 | 38 | 755 | 169 | 1586 | 21.2 |
| BRR085 | 380 | 1752 | 189 | 702 | 29 | 2254 | 25 | 14 | 2322 | 34 | 629 | 128 | 1662 | 18.0 |
| BRR088 | 299 | 1670 | 214 | 920 | 29 | 2131 | 22 | 21 | 2169 | 43 | 851 | 203 | 1548 | 23.4 |
| BRR090 | 416 | 1751 | 178 | 662 | 28 | 2301 | 17 | 15 | 2318 | 38 | 605 | 148 | 1643 | 17.4 |
| BRR091 | 589 | 1717 | 199 | 700 | 26 | 2213 | 24 | 13 | 2232 | 33 | 629 | 154 | 1591 | 18.7 |
| BRR092 | 383 | 1802 | 174 | 678 | 22 | 2231 | 23 | 17 | 2293 | 36 | 682 | 127 | 1652 | 18.1 |
| BRR093 | 871 | 1441 | 265 | 999 | 39 | 1909 | 33 | 24 | 1964 | 39 | 946 | 215 | 1375 | 27.7 |
| BRR095 | 320 | 1677 | 227 | 932 | 32 | 2079 | 31 | 17 | 2126 | 45 | 863 | 194 | 1577 | 23.9 |
| BRR097 | 411 | 1776 | 168 | 658 | 25 | 2276 | 20 | 19 | 2308 | 30 | 579 | 137 | 1713 | 16.9 |
| BRR099 | 364 | 1781 | 168 | 671 | 26 | 2266 | 21 | 16 | 2316 | 34 | 655 | 140 | 1662 | 17.7 |
| BRR101 | 371 | 1735 | 189 | 713 | 25 | 2255 | 20 | 20 | 2317 | 36 | 653 | 139 | 1647 | 18.4 |
| BRR103 | 362 | 1833 | 142 | 559 | 22 | 2329 | 14 | 14 | 2412 | 26 | 566 | 109 | 1732 | 14.9 |
| BRR106 | 338 | 1711 | 213 | 786 | 36 | 2167 | 24 | 22 | 2221 | 43 | 770 | 180 | 1609 | 21.2 |
| BRR107 | 384 | 1818 | 160 | 621 | 27 | 2306 | 17 | 15 | 2360 | 29 | 570 | 136 | 1677 | 16.2 |
| BRR109 | 344 | 1789 | 195 | 748 | 20 | 2223 | 18 | 16 | 2256 | 30 | 681 | 141 | 1659 | 18.9 |
| BRR110 | 353 | 1766 | 181 | 724 | 26 | 2277 | 24 | 19 | 2260 | 32 | 651 | 161 | 1646 | 18.6 |
| BRR111 | 354 | 1750 | 190 | 677 | 28 | 2249 | 19 | 16 | 2341 | 35 | 641 | 155 | 1665 | 18.0 |
| BRR112 | 368 | 1648 | 204 | 826 | 30 | 2181 | 22 | 19 | 2230 | 37 | 813 | 193 | 1549 | 22.0 |
| BRR113 | 345 | 1725 | 196 | 742 | 24 | 2207 | 27 | 18 | 2278 | 35 | 711 | 174 | 1638 | 19.7 |
| BRR116 | 353 | 1794 | 168 | 637 | 25 | 2297 | 23 | 10 | 2368 | 28 | 588 | 132 | 1697 | 16.5 |
| BRR117 | 300 | 1711 | 232 | 837 | 24 | 2146 | 20 | 14 | 2190 | 38 | 821 | 199 | 1588 | 22.3 |
| BRR120 | 355 | 1814 | 161 | 637 | 27 | 2294 | 19 | 14 | 2321 | 30 | 603 | 149 | 1696 | 16.8 |
| BRR122 | 265 | 1702 | 220 | 822 | 36 | 2175 | 28 | 22 | 2230 | 41 | 790 | 168 | 1621 | 21.6 |
| BRR124 | 346 | 1881 | 137 | 529 | 22 | 2316 | 14 | 13 | 2412 | 23 | 513 | 118 | 1796 | 14.0 |
| BRR125 | 368 | 1800 | 165 | 669 | 25 | 2288 | 16 | 15 | 2307 | 28 | 605 | 153 | 1681 | 17.2 |
| BRR126 | 295 | 1781 | 182 | 747 | 36 | 2246 | 20 | 23 | 2289 | 33 | 657 | 147 | 1664 | 18.8 |
| BRR127 | 468 | 1668 | 201 | 728 | 32 | 2226 | 24 | 17 | 2276 | 35 | 717 | 161 | 1567 | 19.8 |
| BRR128 | 301 | 1723 | 208 | 762 | 28 | 2246 | 26 | 19 | 2274 | 37 | 662 | 159 | 1675 | 19.4 |
| BRR129 | 300 | 1739 | 199 | 765 | 29 | 2217 | 18 | 16 | 2268 | 34 | 746 | 180 | 1609 | 20.2 |
| BRR131 | 323 | 1761 | 182 | 731 | 30 | 2238 | 22 | 12 | 2296 | 33 | 682 | 147 | 1663 | 18.8 |
| BRR132 | 577 | 1575 | 209 | 808 | 33 | 2183 | 26 | 18 | 2228 | 38 | 763 | 177 | 1485 | 21.7 |
| BRR133 | 314 | 1748 | 186 | 762 | 22 | 2233 | 24 | 14 | 2283 | 37 | 703 | 167 | 1627 | 19.5 |
| BRR134 | 310 | 1622 | 245 | 926 | 33 | 2142 | 27 | 23 | 2183 | 41 | 811 | 200 | 1557 | 23.5 |
| BRR136 | 280 | 1626 | 247 | 911 | 33 | 2111 | 26 | 26 | 2177 | 44 | 868 | 205 | 1566 | 24.0 |
| BRR138 | 295 | 1723 | 208 | 827 | 28 | 2184 | 22 | 21 | 2207 | 27 | 754 | 183 | 1641 | 21.1 |
| BRR139 | 294 | 1741 | 199 | 767 | 29 | 2220 | 18 | 16 | 2269 | 34 | 745 | 180 | 1608 | 20.2 |
| BRR141 | 320 | 1794 | 193 | 670 | 24 | 2272 | 18 | 13 | 2320 | 33 | 635 | 160 | 1668 | 17.8 |
| BRR142 | 330 | 1751 | 185 | 731 | 24 | 2265 | 17 | 18 | 2300 | 35 | 694 | 145 | 1625 | 18.9 |
| BRR144 | 285 | 1784 | 191 | 708 | 32 | 2273 | 22 | 17 | 2291 | 37 | 650 | 164 | 1666 | 18.5 |
| BRR146 | 319 | 1722 | 187 | 776 | 32 | 2225 | 20 | 21 | 2239 | 38 | 724 | 189 | 1628 | 20.3 |
