# Supplementary material for: Single Nucleotide Polymorphism (SNP) markers associated with high folate content in wild potato species
Source: PLoS One. 2018 Feb 23;13(2):e0193415. doi: 10.1371/journal.pone.0193415 (PMC5825101; doi:10.1371/journal.pone.0193415)
Supplement: S4 Table — Biosynthesis genes are highlighted in blue while salvage and homeostasis genes are highlighted in green. (DOCX) [file pone.0193415.s005.docx]

**S4 Table. Folate metabolism-related genes in Arabidopsis, tomato, and potato.** Biosynthesis genes are highlighted in blue while salvage and homeostasis genes are highlighted in green.

| **Gene name** | **Arabidopsis** | **Tomato** | **Potato** | **Chr.^a^** | **Start Pos.^b^** | **End Pos.** |
| --- | --- | --- | --- | --- | --- | --- |
| GTP cyclohydrolase I (GCHI) | At3g07270 | LOC543831 | PGSC0003DMG400020105 | 6 | 58,218,330 | 58,221,998 |
| Dihydroneopterin (DHN) triphosphate diphosphatase | At1g68760 | Solyc03g043860.2.1 | PGSC0003DMG400030259 | 3 | 6,301,879 | 6,303,243 |
| Dihydroneopterin aldolase (DHNA) | At3g11750  At5g62980  At3g21730 | LOC544263 | PGSC0003DMG400029847  PGSC0003DMG400007623 | 10  4 | 58,006,909  11,351,277 | 58,009,753  11,353,145 |
| Aminodeoxychorismate synthase (ADCS) | At2g28880 | LOC543912 | PGSC0003DMG400009777 | 4 | 32,090,468 | 32,092,714 |
| Aminodeoxychorismate lyase (ADCL) | At5g57850 | LOC778238 | PGSC0003DMG400018587 | 11 | 42,627,656 | 42,632,239 |
| 6-Hydroxymethyldihydropterin pyrophosphokinase (HMDHP-PPK) / dihydropteroate synthase (DHPS) | At4g30000 (mito.)  At1g69190 (cyto., only in Arabidopsis) | Solyc05g012090.2.1 | PGSC0003DMG400028362 | 5 | 692,312 | 694,941 |
| Dihydrofolate synthase (DHFS) | At5g41480 | LOC101257178 | PGSC0003DMG400002352 | 6 | 38,180,979 | 38,187,447 |
| Dihydrofolate reductase (DHFR) | At2g16370 | LOC101267455 | PGSC0003DMG400000736 | 1 | 86,138,599 | 86,145,134 |
|  | At4g34570  At2g21550 |  |  |  |  |  |
| Folylpolyglutamate synthase (FPGS) | At5g05980 (FPGS1) | LOC101250507 | PGSC0003DMG400027193 | 5 | 48,208,214 | 48,215,644 |
|  | At3g10160 (FPGS2) | LOC101246415 |  |  |  |  |
|  | At3g55630 (FPGS3) |  |  |  |  |  |
| UDP-glucose–*p*-aminobenzoate glucosyltransferase | At1g05560 | Solyc12g098590.1.1 | PGSC0003DMG400004573  PGSC0003DMG400004574 | 12  12 | 59,388,655  59,391,505 | 59,391,021  59,393,303 |
| γ-Glutamyl hydrolase (GGH) | At1g78660 (GGH1) | Solyc10g007410.2.1 | PGSC0003DMG400007066 | 7 | 52,454,391 | 52,459,018 |
|  | At1g78680 (GGH2) | Solyc07g062270.2.1 | PGSC0003DMG400021256 | 10 | 2,480,397 | 2,487,536 |
|  | At1g78670 (GGH3) | Solyc07g062280.2.1 | PGSC0003DMG400035974 | 7 | 52,459,355 | 52,462,734 |
| 5-Formyltetrahydrofolate cycloligase (5-FCL) | At5g13050 | LOC543718 | PGSC0003DMG400024570 | 3 | 53,569,914 | 53,573,217 |

^a^ Chromosome number in potato; ^b^ Start and end position in the reference potato genome
